# Induction of IDO1 and Kynurenine by Serine Proteases Subtilisin, Prostate Specific Antigen, CD26 and HtrA: A New Form of Immunosuppression?

**DOI:** 10.3389/fimmu.2022.832989

**Published:** 2022-03-15

**Authors:** Felix I. L. Clanchy, Yi-Shu Huang, Joy Ogbechi, L. Gail Darlington, Richard O. Williams, Trevor W. Stone

**Affiliations:** ^1^ The Kennedy Institute of Rheumatology, Nuffield Department of Orthopaedics, Rheumatology and Musculoskeletal Sciences (NDORMS), University of Oxford, Oxford, United Kingdom; ^2^ Department of Medicine and Rheumatology, Ashtead Hospital, Ashtead, United Kingdom; ^3^ Botnar Research Centre, Nuffield Department of Orthopaedics, Rheumatology and Musculoskeletal Sciences, University of Oxford, Oxford, United Kingdom

**Keywords:** kynurenine, IDO1, Prostate Specific Antigen, PSA, serine proteases, CD26, DPP4, HtrA

## Abstract

Several serine proteases have been linked to autoimmune disorders and tumour initiation although the mechanisms are not fully understood. Activation of the kynurenine pathway enzyme indoleamine-2,3-dioxygenase (IDO1) modulates cellular activity in the brain, tolerogenesis in the immune system and is a major checkpoint in cancer development. We now report that *IDO1* mRNA and IDO1 protein expression (generating kynurenine) are induced in human monocyte-derived macrophages by several chymotryptic serine proteases with direct links to tumorigenesis, including Prostate Specific Antigen (PSA), CD26 (Dipeptidyl-peptidase-4, CD26/DPP-4), High Temperature Requirement protein-A (HtrA), and the bacterial virulence factor subtilisin. These proteases also induce expression of the pro-inflammatory cytokine genes *IL1B* and *IL6*. Other serine proteases tested: bacterial glu-C endopeptidase and mammalian Pro-protein Convertase Subtilase-Kexin-3 (PCSK3, furin), urokinase plasminogen activator (uPA), cathepsin G or neutrophil elastase, did not induce *IDO1*, indicating that the reported effects are not a general property of all serine proteases. The results represent a novel mechanism of activating immunosuppressive *IDO1* and inducing kynurenine generation which, together with the production of inflammatory cytokines, would contribute to tumour initiation and progression, providing a new target for drug development. In addition, the proteasomal S20 serine protease inhibitor carfilzomib, used in the treatment of myeloma, prevented the induction of *IDO1* and cytokine gene expression, potentially contributing to its clinical anti-cancer activity.

## Introduction

The kynurenine pathway, especially the initiating enzyme indoleamine-2,3-dioxygenase-1 (IDO1), is induced in antigen-presenting cells by pro-inflammatory mediators such as interferon-γ (IFN-γ) and bacterial lipopolysaccharides (LPS) (1–3). The pathway has been widely linked to inflammatory and autoimmune disorders (4–9), immune tolerogenesis (10–12) and tumour initiation or maintenance (8, 13–17). IDO1 activity is an important contributor to the prevention and suppression of autoimmune dysfunction since it promotes the differentiation of regulatory T cells (Tregs) and inhibits effector cell function; it is also a major factor in defence against invading micro-organisms and tumour cells (18, 19).

Serine protease activity has similarly been linked with inflammatory and autoimmune disorders including arthritic conditions (20–24) and with the initiation, development, progression and metastasis of a wide range of cancers (25–29). The precise molecular mechanisms underlying these associations remain unclear in most cases although a series of chymotryptic serine proteases down-regulate the tumour suppressor dependence receptors Deleted in Colorectal Cancer, neogenin and unco-ordinated-5 (unc5) (30), leading to a partial epithelial-mesenchymal transition and increased cell migration (31, 32).

In view of these links between serine proteases, kynurenines, inflammation and tumour development, we have examined several proteases for possible interactions with the kynurenine pathway. Representative enzymes from several families of serine proteases were found to play an unexpected role in inducing *IDO1* expression. This is of particular interest because bacterial chymotryptic serine proteases often function as virulence factors which reinforce their proliferative and invasive potential by suppressing the host immune system, in addition to breaking down tissue barriers and cell adhesion molecules. Subtilisin (from *Bacillus subtilis* and other species) has been tested as a bacterial class representative of the large serine protease Clan SB, family S8. Other serine protease virulence factors included glu-C endopeptidase (Glu-C; glutamyl endopeptidase) and High Temperature Requirement protein A (HtrA) although the latter is also produced by mammalian cells. In addition, the mammalian transmembrane T cell activator CD26, which exhibits dipeptidyl-peptidase-4 (DPP-4) serine protease activity and is expressed by many cell types, and Prostate Specific Antigen (PSA) were examined.

The results reveal that *IDO1* gene expression is induced by several tumour-related serine proteases, a phenomenon which may contribute to the suppression of host immunity, the facilitation of bacterial invasion, and oncogenesis. Chymotryptic serine proteases have also been linked with the ability of some viruses to invade host cells, including most recently the SARS-CoV-2 virus (33).

## Materials and Methods

### Reagents

#### Antibodies

Human anti-IDO1 antibody (Clone D5J4E, #86630, Cell Signalling Technology); anti-β-actin antibody (Clone AC-74, #A5316, Sigma); Proteome Profiler Human NFκB Pathway Array (#ARY029, R&D Systems).

#### Proteases

Subtilisin (Type VIII from *Bacillus licheniformis*, #5380, Sigma), human neutrophil elastase (#324681, Sigma), recombinant human CD26/DPP4 (#P1572, Biovision), prostate specific antigen (PSA, #539832, Merck), cathepsin G (#BML-SE283, Enzo Life Sciences Ltd), High Temperature Requirement Protein A (recombinant *E.coli* HtrA, #1670-SE, R&D Systems), cathepsin L (#1515-CY, R&D Systems), chymotrypsin (#7538, Caltag Medsystems Ltd), endopeptidase GluC (Staph. aureus V8, #P8100S New England Biolabs).

#### Inhibitors

N-*p*-tosyl-L-phenylalanine chloromethyl ketone (TPCK, # T4376), N-α-tosyl-L-lysine chloromethyl ketone hydrochloride (TLCK, #90182), suberoylanilide hydroxamic acid (SAHA, #SML0061), anacardic acid (#A7236), decitabine (#A3656), lipopolysaccharide (LPS from *Escherichia coli* O111:B4, #L4391) were purchased from Sigma-Aldrich. Carfilzomib (#S2853) and tocilizumab (#A2012) were purchased from Selleckchem. Ro-106-9920 (#1178), TPCA-1 (#2559), PS1145 (#4569), Bay11-7082 (#1744), Ruxolinitib (#7048) and Stattic (#2798) were purchased from TOCRIS bioscience. TAK-242 (# 614316) was purchased from Millipore. Etanercept was supplied by Pfizer.

### Monocyte-Derived Macrophages

Human apheresis cones were obtained with informed consent from the National Health Service Blood Service (REC: 11/H0711/7). Peripheral Blood Mononuclear Cells (PBMCs) were isolated as previously described (34) using density separation (Lympholyte^®^ *,Cedarlane*). Monocytes were isolated by positive immunomagnetic selection (*Miltenyi*) according to the manufacturer’s instructions (35). Monocytes (10^6^ per mL) were cultured in 10 cm dishes for up to 7 days in complete RPMI 10% with FBS 1%. Penicillin/Streptomycin supplemented with 50 ng/mL of human M-CSF (*Peprotech*) to generate monocyte derived macrophages (MDMs).

### Protease and Inhibitor Treatment

MDMs were detached by gentle scraping and 5 x 10^5^/mL cells were re-plated in tissue culture plates overnight. For protease treatments, MDMs were treated with vehicle, LPS (10 ng/mL), subtilisin (20 nM), Prostate specific antigen (PSA, 100 nM), CD26/DPP4 (10 nM), HtrA (10 nM), Neutrophil elastase (25 μg/mL), Cathepsin G (25 μg/mL), Cathepsin L (25 μg/mL), Chymotrypsin (25 μg/mL) or Endopeptidase Glu-C, all of which were re-suspended according to the manufacturers’ instructions to the highest suggested concentration, then diluted in culture medium before use.

For inhibitor treatments, MDMs were treated with proteases and inhibitors simultaneously overnight. TPCK (30 or 100 μM), TLCK (100 μM) or carfilzomib (20 nM) were added after the proteases whereas SAHA (10 μM), anacardic acid (10 μM), decitabine (2 μM), TAK-242 (10 μM), PS1145 (200 μM), Ro-106-9920 (10 μM), Bay11-7082 (10 μM), TPCA-1 (10 μM), ruxolitinib (20 μM), stattic (10 μM), tocilizumab (20 μg/mL) and etanercept (20 μg/mL) were added before proteases. After treatment, the RNA and protein lysates were extracted for gene and protein expression analysis.

### Gene Expression

RNA extraction was completed according to manufacturer’s instructions (RNeasy Mini Kit, *Qiagen*). A total of 500 ng of RNA was reverse transcribed to cDNA according to the manufacturer’s instructions (High Capacity cDNA Reverse Transcription Kit, *Applied Biosystems*) and diluted to 120 µL (36). Expression of target genes was determined using TaqMan gene expression assays (*ThermoFischer Scientific*) in duplicate using 2.4 μL of cDNA. Gene expression was calculated relative to the housekeeper gene (*HPRT1*) using the δδCT approximation method.

### Protein Expression

Protein lysates were prepared using ice-cold RIPA buffer (*Sigma-Aldrich*). Cells were washed to remove culture medium and cells lysed on ice for 20 min, vortexed and then centrifuged at 17,000 g at 4°C for 20 min. Supernatant was collected and protein concentration measured (BCA protein assay, *Pierce*) (6, 37). IDO1 protein expression was determined after protein gel electrophoresis (NuPAGE Bis-Tris MOPS system, *Invitrogen*) of 10-30 μg of lysate which had been heated to 85°C for 10 min in SDS loading buffer. Proteins were transferred onto PVDF (0.45 μM, *Millipore*) and blocked (5% skim milk powder in 0.05% Tween20/PBS) overnight at 4°C. Blots were stained for hIDO-1 (D5J4E™) or β-actin (AC-74) and detected with HRP conjugated secondary antibodies (P0448 and P0260, *Dako*); after the addition of peroxidase substrate, blots were imaged (G-Box, *Syngene*). Intracellular signalling in response to subtilisin and LPS was determined using a Proteome Profiler™ Array (R&D Systems) according to the manufacturer’s instructions.

### Protease Activity Assay

The activity of proteases was by determined by co-incubation with a substrate (bovine type 2 collagen [bCII] isolated in-house) and then visualised on a protein gel. Briefly, subtilisin was incubated at different concentrations (0-10 μM in 1 μL) or after heating (0-2 h at 70°C) with 5 μg of bCII (5 µL at 1 mg/mL); 4µL of RPMI was added to make a total of 10 µL. After incubation overnight, 2 µL of 6x laemli buffer (*Alfa Aesar*) was added and the digest heated for 5 min at 95°C. After electrophoretic separation of proteins, the gel was washed with de-ionised water and stained with PageBlue™ staining solution (*Thermo Fisher Scientifi*c) for 1 hr, de-stained overnight and digitally imaged (ImageScanner III, *General Electri*c).

### LAL Assay

Protease solutions were prepared with and without LPS and, with or without heating (1 hr at 70°C), were assayed according to manufacturer’s instructions (*Lonza*); the final concentration of exogenous LPS was 10 ng/mL.

### Kynurenine Assay

Kynurenine was measured in cell culture medium as previously described (38). Briefly, samples and standards were added to 30% trichloroacetic acid (T6399, Sigma) at a 2:1 ratio, vortexed, then centrifuged at 700 g for 20 min. Supernatant was added to an equal volume of Ehrlich’s reagent (20 mg/mL p-dimethylaminobenzaldehyde (D2004, Sigma) dissolved in glacial acetic acid) and absorbance read at 492 nm (SPECTROstar Nano, BMG Labtech).

### Statistics

**Figure 1:**
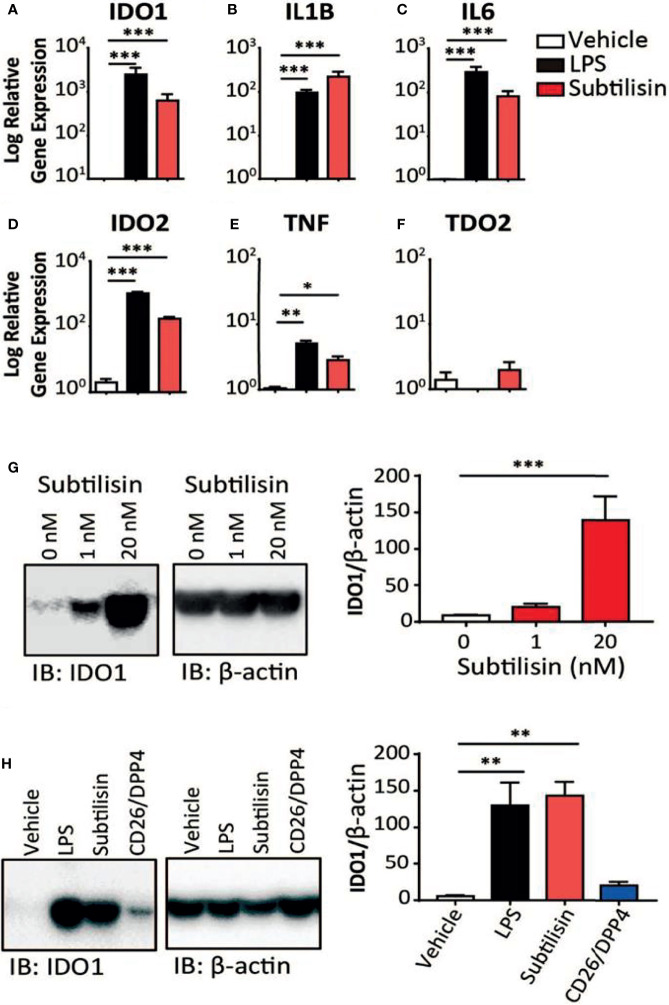
Gene induction by subtilisin. LPS (10 ng/mL) and subtilisin (20 nM; 60 ng/mL) induce the expression of *IDO1* **(A)**, *IL1B* **(B)**, *IL6* **(C)**, *IDO2* **(D)**, *TNF* **(E)** and *TDO2* **(F)** in human monocyte-derived macrophages (MDMs) (logarithmic scale). **(G)** Western blots of MDMs from three different donors shows the expression of IDO1 protein is also induced at 1 nM or 20 nM subtilisin; the bar chart shows the quantified blot density as the ratio of IDO1 to β-actin. **(H)** CD26/DPP4 induces IDO1 but to a lesser extent than LPS or subtilisin; the bar chart shows the quantified blot density as the ratio of IDO1 to β-actin. Charts show the mean ± s.e.m. for (n ≧ 3 donors). The number of experiments (n) was 3 except panels A (n = 18), B (n = 15), C (n = 12) and F (n = 6). *P < 0.05, **P < 0.01; ***P < 0.001.

**Figure 2:**
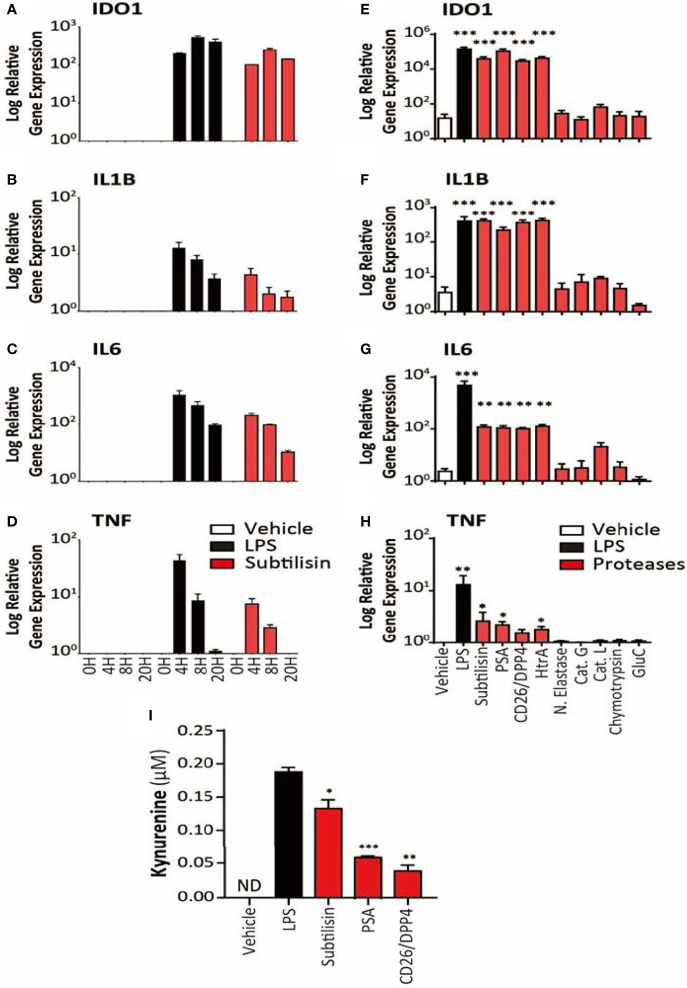
Time course and activity of serine proteases on human MDMs. The left-side panels (logarithmic scales) illustrate **(A)** the apparent maximal induction of *IDO1* after 8 h incubation with LPS (10 ng/mL) or subtilisin (20 nM); **(B)** peak induction before 4 h of *IL1B* and **(C)** IL6; **(D)** a relatively rapid induction of *TNF* expression with a maximum before 4 h declining to baseline by 20 h. The right-side panels indicate the effects of proteases on **(E)** *IDO1* **(F)** *IL1B*, **(G)** *IL6* and **(H)** *TNF* expression by vehicle, LPS (10 ng/mL), subtilisin (20 nM), PSA (100 nM), CD26/DPP4 (10 nM), HtrA (10 nM), neutrophil elastase (25 μg/mL), cathepsin G (25 μg/mL), cathepsin L (25 μg/mL), chymotrypsin (25 μg/mL) and glu-C (10 nM). Panel **(I)** shows the concentration of kynurenine in the culture supernatant of cells exposed to LPS, subtilisin, PSA and CD26/DPP4. Charts show the mean ± s.e.m. (n ≧ 3 donors). The number of experiments (n) was 3 (for A-D) or n ≧ 3 (for E-I). *P < 0.05, **P < 0.01; ***P < 0.001.

**Figure 3:**
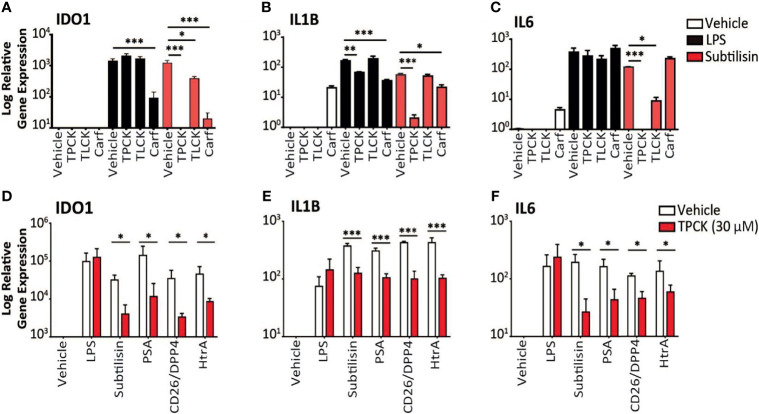
Inhibition of serine protease activity. **(A–C)** The charts illustrate the expression relative to baseline (logarithmic scale) of **(A)** *IDO1*, **(B)** *IL1B*, **(C)** *IL6* and the effects of vehicle (open bars), 10 ng/mL LPS (black bars) or 20 nM subtilisin (red/shaded bars). The inhibitors tested were TPCK (100 μM), TLCK (100 μM) and carfilzomib 20 nM (Carf). **(D–F)** The charts show a comparison of the effects of TPCK (30 μM) on the induction of *IDO1* **(D)**, *IL1B* **(E)** and *IL6* **(F)** by LPS (10 ng/mL), subtilisin (20 nM), PSA (100 nM), CD26/DPP4 (10 nM) and HtrA (10 nM). Expression is shown for vehicle (open bars) and TPCK (30 μM) (red/shaded bars) treatments. Charts show the mean ± s.e.m. (n ≧ 3 donors). *P < 0.05, **P < 0.01; ***P < 0.001.

**Figure 4:**
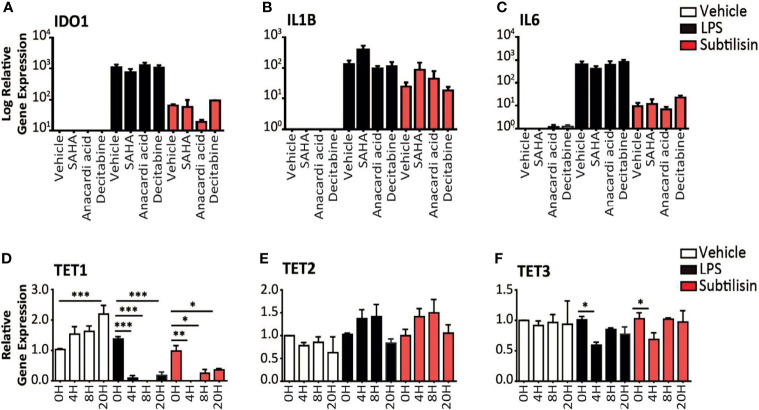
Epigenetic modulators and gene expression. **(A–C)** Charts of the gene expression (logarithmic axis) relative to baseline of **(A)** *IDO1*, **(B)** *IL1B* and **(C)** *IL6* in MDMs exposed to vehicle (open bars), 10 ng/mL LPS (black bars) or 20 nM subtilisin (red/shaded bars). The data shows the effects of 10 μM SAHA (an inhibitor of histone deacetylase), 10 μM anacardic acid (an inhibitor of histone acetylase) and 2 μM decitabine (an inhibitor or DNA methyltransferase). **(D–F)** Logarithmic expression relative to baseline of TET1 **(D)**, TET2 **(E)** and TET3 **(F)** in cells exposed to vehicle (open bars), 10 ng/mL LPS (black bars) or 20 nM subtilisin (red/shaded bars). Charts show the mean ± s.e.m. (n ≧ 3 donors). *P < 0.05, **P < 0.01; ***P < 0.001.

**Figure 5:**
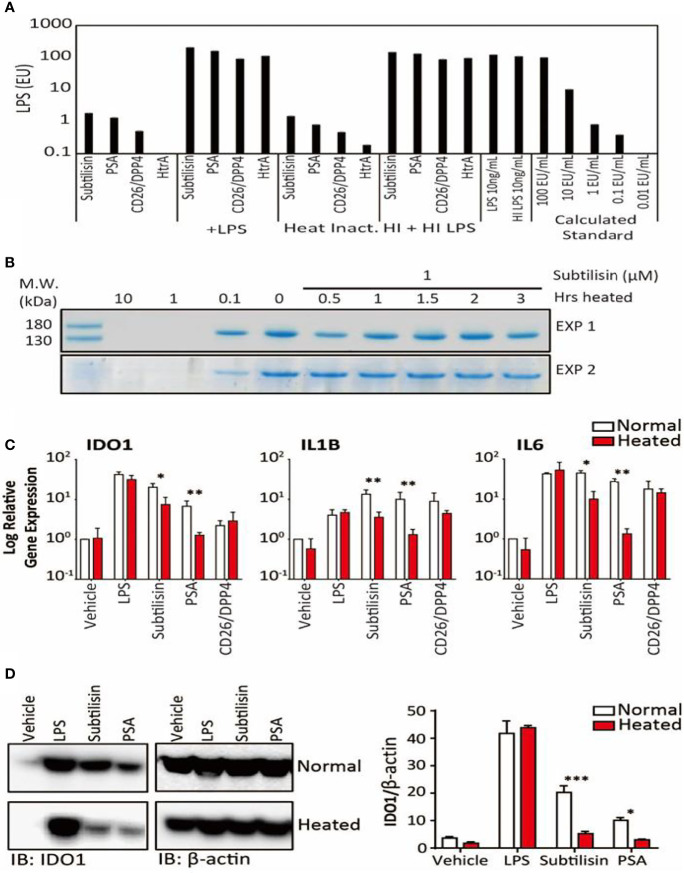
LPS concentrations and the effects of heating on gene expression. **(A)**. Samples of the four active serine proteases were analysed for endotoxin contamination. In bars 1-4, when compared against LPS 10 ng/mL, subtilisin (20 nM) is associated with < 1% LPS, while PSA (100 nM), CD26/DPP4 (10 nM) and HtrA (10 nM) have even lower amounts. Bars 5-8 show that the serine proteases do not affect the LPS signal, confirming that they do not interfere with the assay procedure. Bars 9-12 and 13-16 reveal that heating the proteases at 70°C for 1 h did not alter the protease signals (9–12) or the combined protease and LPS signals (13–16), confirming that LPS was not affected by the heat treatment and was still not affected by the proteases. This conclusion is strengthened by the absence of heat on LPS alone (bars 17-18). Bars 19-23 show a calibration for the absolute concentration of LPS in this assay. **(B)** A Coomassie Blue stained gel of collagen in two separate experiments shows that the undigested protein in the absence of subtilisin (0 μM) is reduced at 0.1 μM and abolished at 1 and 10 μM. After heating subtilisin at 70°C for 0.5 h or longer, protease activity has been substantially reduced and is not able to degrade the collagen. **(C)** Quantified data for the heating experiments. Bar charts show the relative expression of genes *IDO1*, *IL1B* and *IL6* by LPS (10 ng/mL), subtilisin (20 nM), PSA (100 nM) and CD26/DPP4 (10 nM) under normal experimental conditions (open bars) and after heating at 70°C for 1 h (red/shaded bars). Heating removes much of the activity of subtilisin and PSA on all three genes. **(D)** Representative Western blot shows that the more heat-sensitive proteases (20 nM subtilisin and 100 nM PSA) induce less IDO1 protein when heated at 70°C for 1 h. The bar chart shows the quantified ratio of IDO1 to β-actin density. Charts show the mean ± s.e.m. (n ≧ 3 donors). *P < 0.05, **P < 0.01; ***P < 0.001.

**Figure 6:**
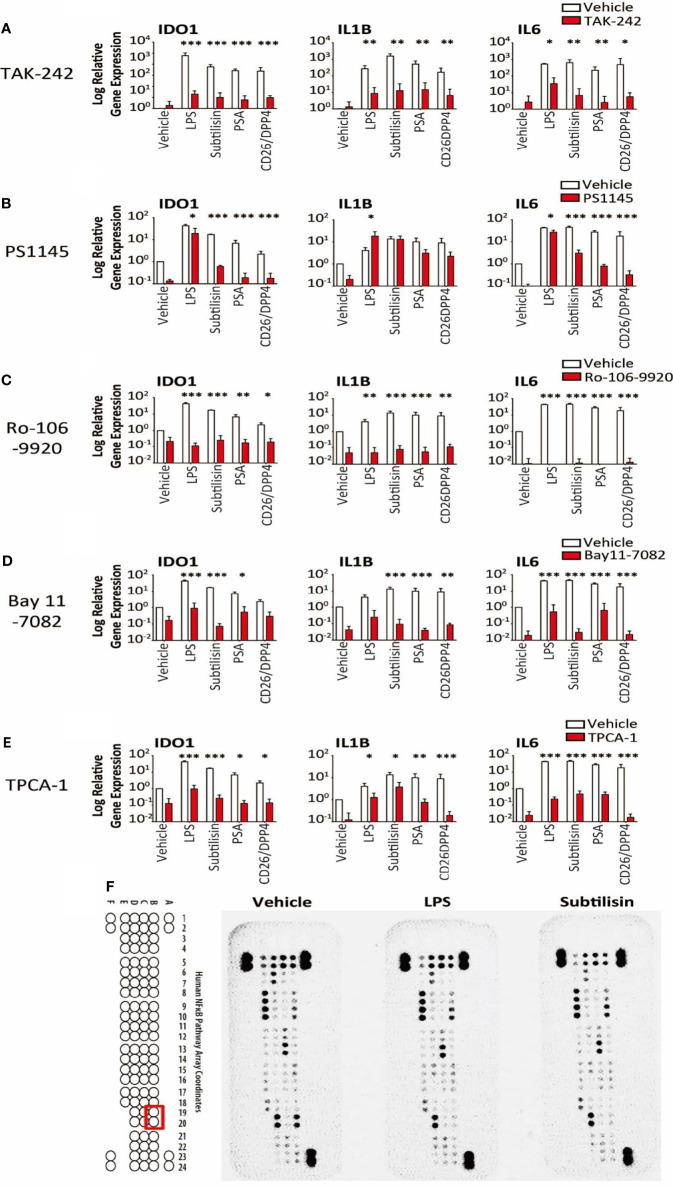
Transduction pathways affected by serine proteases. The logarithmic relative expression of *IDO1*, *IL1B* and *IL6* induced by vehicle, LPS (10 ng/mL), subtilisin (20 nM), PSA (100 nM) and CD26/DPP4 (10 nM) in the presence of vehicle (open bars) or test agents (red/shaded bars): **(A)** TAK-242 (10 μM), **(B)** PS1145 (200 μM), **(C)** Ro-106-9920 (10 μM), **(D)** Bay11-7082 (10 μM) or **(E)** TPCA-1 (10 μM). Charts show the mean ± s.e.m. (n ≧ 3 donors). *P < 0.05, **P < 0.01; ***P < 0.001. **(F)** illustrates a proteomics array based on NFκB transduction pathways exposed to vehicle, LPS (10 ng/mL) or subtilisin (20 nM) for 0.5 h. LPS and subtilisin induced a loss of B19 and B20 corresponding to IκBε. Bars show the mean ± s.e.m. (n ≧ 3). *P < 0.05, **P < 0.01; ***P < 0.001.

**Figure 7:**
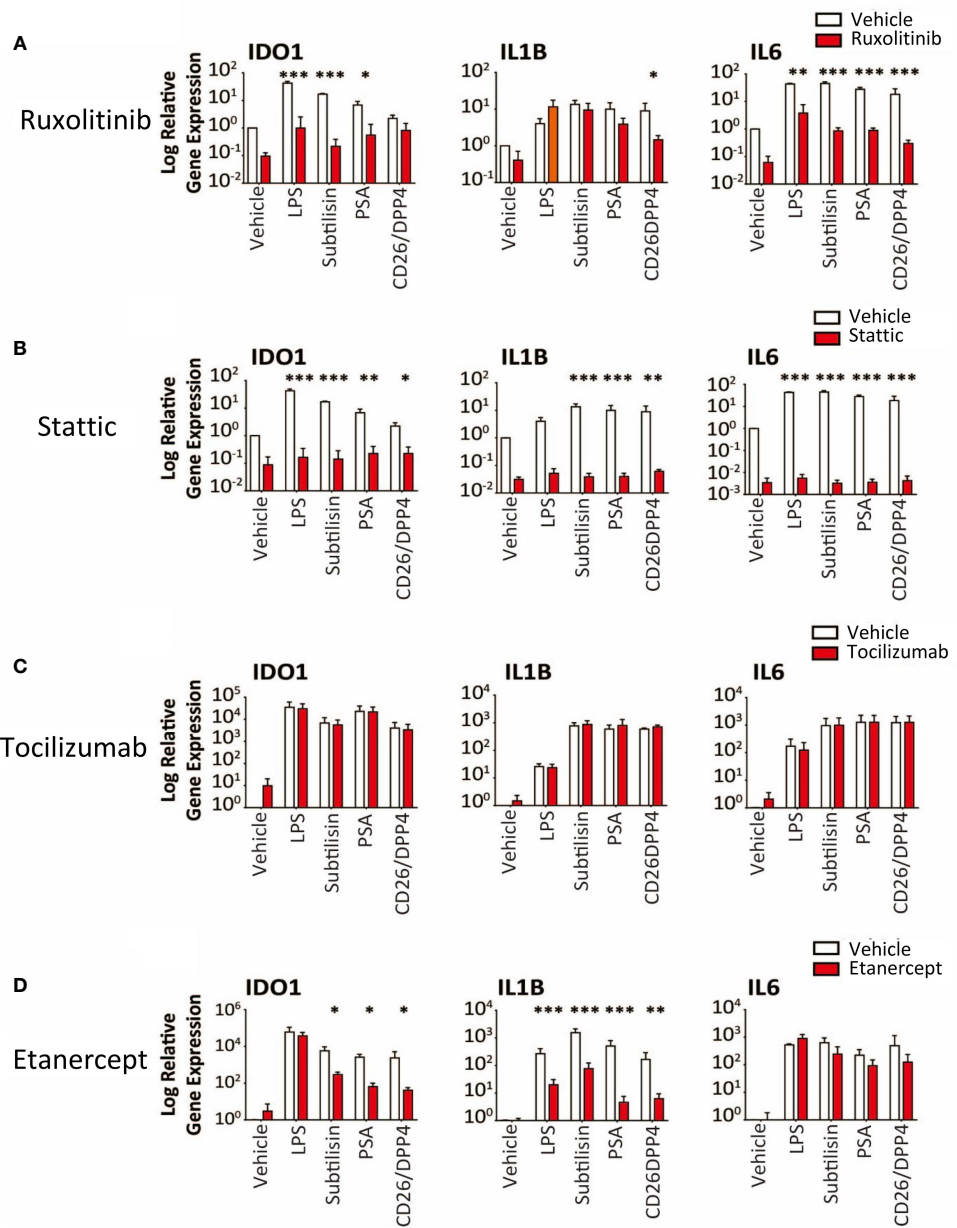
Transduction pathways affected by serine proteases. The logarithmic expression of *IDO1*, *IL1B* and *IL6* induced by vehicle, LPS (10 ng/mL), subtilisin (20 nM), PSA (100 nM) and CD26/DPP4 (10 nM) in the presence of vehicle (open bars) or test agents (red/shaded bars): **(A)** Ruxolitinib (20 μM), **(B)** stattic (10 μM), **(C)** tocilizumab (20 μg/mL), **(D)** etanercept (20 μg/mL). Bars show the mean ± s.e.m. The number of experiments (n) was 3 in all cases except panels C and D, where n = 6. *P < 0.05, **P < 0.01; ***P < 0.001.

Vehicle controls were included for all experiments, except **Figure 2I** proteases vs. LPS and **Figures 5C, D** normal *vs.* heated. One-way ANOVA with Tukey’s multiple comparison test with a single pooled variance was performed for statistical comparisons of **Figure 1**, **Figure 2**, **Figures 3A–C** and **Figure 4**. Two-way ANOVA with Tukey’s or Sidak’s multiple comparison test was used for statistical comparisons of **Figures 5C, D**, **Figures 3D–F**, **Figure 6** and **Figure 7**, as appropriate. All calculations were performed using GraphPad Prism 7 software. A ‘P’ value less than 0.05 was considered significant

## Results

### Serine Proteases Induce IDO1

When human monocyte-derived macrophages were exposed for 24 h to LPS (10 ng/mL) the expression of *IDO1* was increased compared with unstimulated cells (**Figure 1**). At a concentration of only 20 nM (60 ng/mL), the bacterial protease subtilisin induced *IDO1* expression to a similar extent (**Figure 1**). Both agents also induced *IL1B* and *IL6* expression, although subtilisin was 5-fold more potent on *IL1B* (**Figure 1**) and 5-fold less potent on *IL6* (**Figure 1**). Both ligands induced the expression of *IDO2* (**Figure 1**) and *TNF* (**Figure 1E**), although the latter was induced to a much lesser degree (<10-fold). No significant induction of *TDO* was observed (**Figure 1F**).

To assess whether the induction of *IDO1* was translated into protein expression, Western blots were performed on macrophage extracts. Exposure to subtilisin at 1 nM and 20 nM was sufficient to generate a marked, concentration-related expression of IDO1 protein (**Figure 1G**). When repeated to compare subtilisin with LPS and CD26/DPP4, a strong induction of IDO1 was produced by LPS and subtilisin, while CD26/DPP4 also induced a clear IDO1 protein signal, although weaker in intensity (**Figure 1H**). PSA also induced IDO1 as reported below (**Figures 5C, D**).

An examination of the time course indicated a maximal response of *IDO1* to LPS or subtilisin after 8 h (**Figure 2A**). A more rapid time course, peaking at or before 4h was observed for *IL1B* (**Figure 2B**), *IL6* (**Figure 2C**) and *TNF* (**Figure 2D**) although the latter was relatively transient and declined to control levels by 20 h.

In view of the marked induction of *IDO1* by subtilisin, the experiments were extended to a number of chymotryptic serine proteases related to subtilisin and some of which have been associated with the induction of tumour properties in cells. **Figure 2** shows the similar degree of induction of *IDO1* expression by subtilisin and CD26/DPP4 but shows a comparable activity by PSA and HtrA, a membrane serine protease expressed by mammalian cells and by bacteria as a potent virulence factor. In contrast, several other serine proteases were not active relative to controls, including neutrophil elastase, cathepsin G, cathepsin L, chymotrypsin and the bacterial virulence factor glu-C endopeptidase (Glu-C) (**Figure 2E**). A similar pattern was seen on cytokine induction where subtilisin, CD26/DPP4, PSA and HtrA induced large increases in *IL1B* (**Figure 2F**) and *IL6* (**Figure 2G**) with lower, but statistically significant expression of TNF (**Figure 2H**).

### Kynurenine Production

To determine whether the IDO1 gene induction was translated into IDO1 protein with enzymic activity, cells were incubated with vehicle, LPS, subtilisin, CD26/DPP4 or PSA. No kynurenine was detected in the unstimulated culture supernatants, but the results clearly show an increase in kynurenine production by the proteases as shown as **Figure 2I**. The generation of kynurenine broadly reflected the levels of IDO1 protein expression, with subtilisin being the most active protease, CD26/DPP4 being the least active (see **Figure 1H**) and with intermediate activity of PSA (see **Figure 5D**).

### Elimination of Endotoxin Contamination as a Cause of IDO Induction

In view of the potency of the active proteases, experiments were designed to exclude the possibility of contamination. Firstly, samples of the active enzymes subtilisin, PSA, CD26/DPP4 and HtrA were subjected to the Limulus Amoebocyte Lysate (LAL) test (**Figure 5A**). Subtilisin gave a signal equivalent to ~1% that of 10 ng/mL of LPS, a concentration commonly used to induce maximal gene expression of inflammatory mediators. Even lower signals were obtained for PSA or CD26/DPP4 and none in HtrA.

Secondly, subtilisin was heated at 70°C for between 0.5 and 3 h before testing for enzymic activity. **Figure 5B** shows that 0.1 μM had clear enzymic activity (reducing collagen concentration), supporting the routine use of concentrations up to that level. It was also shown that heating for 0.5h was sufficient to substantially inhibit the activity of subtilisin at the tested concentration of 1 μM, with complete inhibition measured after 1 h heat inactivation. The ability of PSA to induce *IDO1* was also reduced by heating (**Figure 5D**). Quantifying similar experiments for LPS, subtilisin, PSA and CD26/DPP-4 confirmed that across the three genes LPS activity was unaffected by heating (**Figure 5C**), subtilisin activity was reduced by 65-75%, while PSA activity was almost abolished (**Figure 5C**). The limited inhibition of subtilisin on gene expression may reflects its resistance to heating, as some forms of subtilisin retain activity after 95°C heat (39) and show optimal activity at 65°C (40). The effect of heating LPS using the same temperatures as used for proteases had little effect on measurement by LAL assay (**Figure 5A**), supporting its stability under the test conditions and that it could not have been responsible for the activity of subtilisin or PSA. In order to eliminate the possibility that these results could have been caused by the serine proteases interfering with the assay (for example, by metabolising the proteins involved), we combined the test enzymes with LPS, showing that the LPS signal was no different from its control level (**Figure 5A**).

Surprisingly, gene induction by CD26/DPP-4 was not changed significantly by heating (**Figure 5C**). However, previous studies of its thermostability have placed this protein among those which are relatively heat-resistant; indeed, the optimal temperature for CD26/DPP4 activity is approximately 50°C with around 35% of activity remaining after 60°C (41–43).

Thirdly, the proteases were tested in the presence of the selective chymotryptic serine protease inhibitor N-alpha-tosyl-L-phenylalanyl chloromethylketone (TPCK). At the widely used concentration of 100 μM this compound had no effect on *IDO1* induction by LPS, but prevented induction by subtilisin (**Figure 3A**). The related compound N-alpha-p-tosyl-L-lysine chloromethyl ketone (TLCK, 100 μM), which acts primarily on enzymes with a more trypsin-like substrate specificity also had no effect on the induction of *IDO1* by LPS and only partially (approximately 50%) inhibited subtilisin (**Figure 3A**). This was consistent with the view that subtilisin is primarily a chymotryptic protease with some overlap in its substrate specificity. A similar overall profile of inhibitory activity was observed for *IL1B* expression (**Figure 3B**). TPCK also fully inhibited the effect of subtilisin on IL6 induction, with no effect on the response to LPS, (**Figure 3C**). TLCK was again less effective than TPCK but reduced subtilisin-induced *IL6* expression by an order of magnitude (**Figure 3C**), consistent with subtilisin acting independently of LPS. Partly to clarify this result and partly to extend the experiment, this work was repeated using a lower concentration of TPCK (30 μM) and comparing LPS with subtilisin, PSA, CD26/DPP4 and HtrA (**Figure 3D** **)**. While reduction of the serine protease induction of *IDO1* was correspondingly less than observed previously with 100 μM, TPCK at 30 μM still reduced serine protease activity by 2-3 orders of magnitude (**Figure 3D**). Importantly, the effect of LPS was unaffected at that concentration. Induction of *IL1B* behaved similarly to *IDO1* (**Figure 3E**), while *IL6* expression by the proteases was more sensitive to TPCK (**Figure 3F**). The absence of any effect of TPCK on LPS suggests that there were no issues with non-specific or toxic actions of the chloromethylketones. Overall, this extended experiment fully confirmed the predominant role of serine protease activity in the induction of *IDO1*, *IL1B* and *IL6* by subtilisin, PSA, CD26/DPP4 and HtrA.

### Proteasomal Serine Protease Inhibitor Carfilzomib Blocks IDO1 Induction

Carfilzomib is an inhibitor of the S20 chymotryptic module of the ubiquitin-related proteasome, a selectivity which underlies its use in the treatment of multiple myeloma (44). Carfilzomib was included, therefore, in the expectation that it would not affect the panel of serine proteases used in this work. However, carfilzomib (20 nM) was able to substantially reduce the induction of *IDO1* by subtilisin (**Figure 3A**), with a small but significant reduction of *IL1B* expression (**Figure 3B**) but no effect on *IL6* expression (**Figure 3C**). The same pattern was observed using LPS, with carfilzomib inhibiting the induction of *IDO1* (**Figure 3A**), a small effect on *IL1B* (**Figure 3B**) but not *IL6* (**Figure 3C**). The cytokine results are consistent with their differential sensitivity to a systemic and proteasomal inhibitor, while the *IDO1* induction strongly suggests a greater pharmacological overlap between the proteasomal chymotryptic activity and that of a bacterial serine protease such as subtilisin. The suppression of *IDO1* induction by carfilzomib could enhance its therapeutic activity in myeloma, a problem potentially exacerbated by the apparent ability of carfilzomib alone to induce some *IL1B* and *IL6* expression (**Figures 3B, C**).

### Mechanisms of Action: Epigenetic Factor Are Not Involved

In order to determine the mechanism of *IDO1* induction by serine proteases we considered the possible proteolytic removal of a transcription inhibitor affecting methylation or acetylation. Gene expression can be optimised by the arrangement of associated histone proteins, acetylation of which (by histone acetylase, HAC) maintains their normal organisation. Histone deacetylases (HDAC) remove this stabilisation and inhibit gene expression, so inhibitors of HDAC would maintain acetylation and promote *IDO1* expression. Incubation with the HDAC inhibitor SAHA did not significantly affect the expression of *IDO1* (**Figure 4A**), *IL1B* (**Figure 4B**) or *IL6* (**Figure 4C**). Anacardic acid (an inhibitor of HAC) had no significant effect on the induction by subtilisin of *IDO1* (**Figure 4A**) *IL1B* (**Figure 4B**) or *IL6* (**Figure 4C**).

Proteolytic interference with DNA methyltransferase (DNMT), reducing DNA methylation, can increase *IDO1* gene expression (6). However, when macrophages were exposed to decitabine, an inhibitor of DNMT, there was no change in the expression of *IDO1* (**Figure 4A**), *IL1B* (**Figure 4B**) or *IL6* (**Figure 4C**) in cells exposed to LPS or subtilisin. As an alternative approach to examine the possible involvement of methylation, expression of the TET (Ten-Eleven-Translocation) enzymes were examined, since they promote oxidation of methyl-cytosine, facilitating both active and passive gene demethylation. The expression of TET1 increased progressively in control cells up to 20 h of incubation (**Figure 4D**). Neither LPS nor subtilisin had any effect on baseline levels but TET1 expression at all subsequent time points was substantially reduced to very low levels compared with the controls (**Figure 4D**). TET2 expression showed no significant changes, with only a limited trend to increase (**Figure 4E**). The expression of TET3 exhibited only a limited but significant inhibition of expression at 4 h (**Figure 4F**).

### Mechanisms of Action: IDO1 Induction Is *via* a TLR4 Pathway

LPS is thought to exert most of its activity through Toll-Like Receptor-4 (TLR4) (45) and TAK-242 (CLI-095) has been reported to be a selective inhibitor (46). Inductions of *IDO1, IL1B* and *IL6* by the proteases were all reduced significantly by TAK-242 (**Figure 6A**), consistent with an involvement of TLR4 in the induction of all these genes.

It is known that *IDO1* can be induced *via* the JAK/STAT1/3 pathway and NFκB. The direct NFκB inhibitor PS1145 (N-(6-chloro-9 H-b-carbolin-8-yl) nicotinamide β-carboline; 200 μM) reduced the gene induction by LPS and serine proteases, although the effects of LPS were least affected (**Figure 6B**). As LPS stimulation leads to NFκB signalling, the variation in these results was surprising and additional, selective inhibitors were examined. Both Ro-106-9920 (10 μM) (**Figure 6C**) and BAY11-7082 (10 μM) (**Figure 6D**) were able to suppress very clearly the responses to LPS in addition to subtilisin, PSA and CD26/DPP4. Involvement of the NFκB system was further supported by the use of TPCA-1 (2-(amino-carbonyl)-amino-5-(4-fluorophenyl)-3-thiophenecarboxamide), an inhibitor of IκB-kinases, key regulators of NFκB activation. At a concentration of 10 μM this compound reduced the induction of *IDO1* by all the test agents, including LPS, subtilisin, PSA and CD26/DPP4 by between 25-50% (**Figure 6E**). In view of these results a proteomics array was used covering 100 molecules associated with NFκB activation to examine possible changes induced by exposure to LPS or subtilisin (10 nM) for 30 min. This procedure affected the expression of only one of the assayed compounds, IκBε, with no other changes being detected (**Figure 6F**, spots B19, B20). Since IκBε is a component of the pathways regulating NFκB activity, this result is entirely consistent with the inhibitory effects of the direct NFκB inhibitors, while indicating the involvement of a specific element of those pathways with no change in the more commonly affected IκBα and IκBβ.

Ruxolitinib (10 μM) is an inhibitor of JAK1/2 but had variable activity, inhibiting induction of *IDO1* by subtilisin and PSA, but not CD26/DPP4 (**Figure 7A**). In direct contrast, only CD26/DPP4 was prevented from inducing *IL1B*, while the induction of *IL6* by LPS and all the proteases was blocked by ruxolitinib (**Figure 7A**). Finally the STAT3 inhibitor stattic (10 μM) produced a substantial inhibition of *IDO1*, *IL1B* and *IL6* induction by LPS, subtilisin and PSA (**Figure 7B**).

Overall, the results indicated clearly that the serine protease induction of IDO1 and cytokines involved the activation of NFκB, at least partly *via* IκBε and STAT3. However, the induction of *IL1B* and *IL6* raised the possibility that these, and other, cytokines, once released into the medium, could have acted in an autocrine or paracrine fashion on those cells and affected their sensitivity to the test ligands and their interaction with NFκB. This was deemed unlikely since inclusion of the IL-6 receptor antibody tocilizumab at 5 or 20 μg/mL (**Figure 7C**) had no effect on the results although the anti-TNF compound etanercept (20 μg/mL) did reduce the effects of the proteases on *IDO1* and *IL1B* induction, with no change of *IL6* expression (**Figure 7D**). This suggests that TNF may act as an intermediary contributing to the activation of IκBε and STAT3 in this work.

## Discussion

The results of this study show that several mammalian and bacterial enzymes with serine protease activity induce *IDO1* in monocyte-derived human macrophages. These are among the first cells of the innate immune system to detect invading microorganisms or cells which might lead to tumour initiation (31, 34, 44). Serine proteases are a normal product of leucocyte activity, inactivating and destroying foreign, damaged or mutated cells. They are often released from cancer cells to promote tumour progression and metastasis by suppressing host immune responses (47–49). Conversely a number of serine protease inhibitors, including TPCK and TLCK used here, suppress tumour development, cell migration and metastasis (49–51). The levels of endogenous serine protease inhibitors are often subnormal in cancer development and progression (52).

The kynurenine pathway (1, 3, 53) functions as an important regulator of immune system activity (12, 14, 18, 54, 55) generally suppressing inflammatory activity. This is achieved by promoting Treg production partly by activation of General Controller Non-derepressible-2, producing inhibition of cell proliferation (56) and partly by generating kynurenine and 3-hydroxyanthranilic acid (3HAA) (25, 57, 58). Kynurenine regulates the balance between Th17 effector and Treg cells (59), *via* aryl hydrocarbon receptors (AHRs) and their induction of IDO and TDO (15). This establishes a positive feedback generation of kynurenine which induces expression of the Treg -generating transcription factor FoxP3 and inhibits the Th17 inducer Retinoic Acid Receptor-related Orphan Receptor-γt (15, 60, 61). The downstream metabolite 3HAA shifts the pro-inflammatory and anti-inflammatory balance directly by inhibiting Th1 cell differentiation and function (58) and may also influence the expression of nuclear transcription co-activators (61). Microbial invasion and tumour aggression are therefore facilitated by the expression of *IDO1* and its suppression of host immune surveillance and cell destruction (62, 63), coupled with suppression of the differentiation and activity of effector T cells and NK cells (16, 19, 64–66).

Prostate Specific Antigen (PSA) has been used as a biomarker for prostate cancer since its blood levels are often elevated in affected patients. Its use is limited by the large variance in these concentrations and the high rates of false positive or negative results. Nevertheless, PSA alone can be a reliable marker of specific aspects of disease progression or recurrence (67). In addition, modified measurements such as free and total PSA, or the rate of change over time are more useful. The levels of PSA together with metabolically related molecules such as kallikreins, or endogenous derivatives and complexes of PSA have also proved more reliable than PSA alone. The development of PSA as the basis of imaging and the development of algorithms for integrating features of selectivity, tumour stage and time course, can provide substantial improvements in diagnosis and assessment (68–71).

CD26 exhibits DPP-4 serine protease activity and is a member of the Type2-transmembrane protease family. Cytotoxic T-lymphocytes are the primary source of soluble CD26 (sCD26), and their activation promotes CD26/DPP-4 translocation to the cell surface from where it is released (72). It is widely expressed in myeloid derived cells (72–74) somatic muscle cells, adipocytes, epithelial and endothelial cells where it can act as a plasminogen receptor (75). CD26/DPP-4 is targeted by inhibitors for the treatment of type-2 diabetes mellitus (76), Crohn’s disease (77) and other inflammatory or autoimmune conditions (72, 73, 76, 78–81) and has been implicated in a wide variety of cancers (82–85). The induction of *IDO1* by CD26/DPP-4 demonstrated here may contribute to many of these disorders.

The HtrA serine proteases are major microbial virulence factors which break down host extracellular and adhesion molecules to increase migration rate and tissue invasion (86–89). HtrA is one of the most important products of *Helicobacter pylori*, causing severe inflammation, damage to the gastric mucosa (90) and gastric cancer in around 3% of infected patients (91). Expression of HtrA proteins is associated with tumour initiation (92–94) and the induction of *IDO1* reported here may contribute to these actions, a result which may be linked to the induction of HtrA1 by TGF-β, a prominent inducer of IDO expression. Expression of HtrA1 is also increased in experimental arthritis and may be involved in this condition (95).

Subtilisin is released as a virulence factor by *B. subtilis* and other species and is used widely in commercial and domestic cleaning products as well as in the tenderising of meats and other processed food products. The ability of subtilisin to induce IDO at low nanomolar concentrations may therefore be relevant to the effects of environmental activity and diet in promoting susceptibility to oncogenesis.

Although the emphasis in this study has been on gene induction, the increase in IDO1 expression was translated into IDO1 protein. It was demonstrated that the protein was enzymically active by the production of kynurenine measured in the culture supernatant. This is important since IDO1 can also have non-enzymatic activity which contributes to the regulation of T cell tolerance *via* the recruitment of Transforming Growth Factor-β (11, 96).

In explaining the mechanisms for the serine protease induction of IDO1, the potential role of histone acetylation in the kynurenine pathway is indicated by the finding that histone deacetylase Hst1 regulates the *de novo* synthesis of NAD, the final product of the pathway. Incubation with the HDAC inhibitor SAHA reduced the expression of *IDO1* and *IL6* in resting, untreated cells (97), although it also tended to increase expression of *IL1B* by LPS or subtilisin. However, anacardic acid, an inhibitor of HAC, reduced the induction of IDO1 by subtilisin as would be predicted from reduced acetylation. Overall, the pattern of results is not consistent with a clear, simplistic role of histone acetylation in the induction of *IDO1* by serine proteases. Different roles of histone acetylation may apply to different stages in *IDO1* induction or may differentially involve STAT1/3 activity (98, 99). Butyrate may reduce IDO expression partly by inhibiting STAT1 and partly by inhibiting HDAC (100, 101).

The results suggest that the canonical NFκB activation pathway is involved in the serine protease activity, as the NFκB inhibitors PS1145, Ro-106-9920 and BAY11-7082 inhibited their activity, as did the JAK1/2 inhibitor ruxolitinib. Furthermore, TPCA1 and stattic STAT3 were highly effective inhibitors. This raised the possibility that a canonical pathway might be involved in the protease actions, a view supported by the loss of IκBε expression, a prominent component of canonical activation of NFκB. The activation of a non-canonical pathway to NFκB has been invoked previously to explain some effects of *IDO1* induction (102).

An important additional conclusion from the proteomics analysis is that the serine proteases did not induce a widespread alteration of cell signalling, consistent with the serine proteases having a selectivity of action on IDO1 expression rather than, for example, inducing a generalised change in cell proliferation or phenotypic differentiation.

Carfilzomib alone induced the expression of IL1B and IL6, a result which may indicate inhibition of a proteasomal enzyme capable of modulating gene expression. More surprising was its inhibition of IDO1 and *IL1B* induction by LPS or subtilisin, the IDO1 suppression being very marked with changes of 1 to 2 orders of magnitude. It is possible that carfilzomib is inhibiting the generation of a protease-dependent intermediate factor, which contributes to the induction of IDO1. An example from this study might be TNF, blockade of which reduces IDO1 induction, and the soluble form of which is itself generated by proteolytic activity of TNF Alpha Converting Enzyme. Carfilzomib is a second generation, irreversible inhibitor of chymotrypsin-like activity in the 20S proteasome in most cells (103, 104) and is used in the treatment of multiple myeloma (105). It is generally considered that there is little substrate or inhibitor overlap between non-proteasomal and S20 chymotryptic enzymes, although these do enter the circulation after cell damage where levels are positively correlated with the symptoms of multiple myeloma and with the disease stage (106). Therefore, it is possible that part of the therapeutic success of carfilzomib lies in its ability to reach all cell types in the blood and tissues where it may inhibit *IDO1* expression, contributing to its anti-cancer efficacy.

The profile of cytokine induction is different for the various enzymes. While TLR4 is in important mediator of serine protease activity we suggest that the results reveal the ability of different ligands to induce distinct transduction routes from a common receptor, a phenomenon originally postulated for the bacterial protein tenascin-C and its fragments, also acting on TLR4 (107, 108). Factors affecting this conclusion might include the different balance of extracellular and intracellular penetration and activity, and the selective, or relative, binding to components of the receptors, since TLR4 is dependent on the associated CD14 and MD-2 co-receptors. In principle this conclusion raises the important concept that drugs interfering with different epitopes on a receptor may have quite different clinical relevance and therapeutic potential. Overall, however, the induction of IDO1 and pro-inflammatory cytokines by pro-carcinogenic serine proteases may represent a novel, additional mechanism of immunosuppression by invading microbes and tumour cells, raising the possibility that they should be considered targets for new drug development.

## Conclusion

Our data indicate that four serine proteases associated with carcinogenesis can act on TLR4 to induce the expression of *IDO1* and pro-inflammatory cytokine genes. The receptor-activated pathways are not the same as those entrained by LPS, although there are common features. Since *IDO1* is a key factor in immune system tolerogenesis and immune privilege, and high levels of IDO1 activity are associated with a poor prognosis for many cancers, especially solid tumours, its induction by serine proteases may contribute to the suppression of host immunity, the facilitation of bacterial invasion and carcinogenesis. This is clearly important for host genes such as PSA and CD26/DPP4, but prokaryotic serine proteases such as subtilisin are present in soil and are frequently used in cleaning materials, meat tenderisation and food processing. There may therefore be wider implications for the relationship between environment and health.

## Data Availability Statement

The raw data supporting the conclusions of this article will be made available by the authors, without undue reservation.

## Ethics Statement

The studies involving human participants were reviewed and approved by National Health Service Blood Service (REC: 11/H0711/7). The patients/participants provided their written informed consent to participate in this study.

## Author Contributions

FC, TS, and RW initiated the project and designed the study. Y-SH and FC conducted the experimental work. All authors (TS, RW, FC, Y-SH, JO, and LD) contributed to writing and reviewing the manuscript. All authors contributed to the article and approved the submitted version.

## Funding

This project was supported in part by funding from the CRUK Oxford Centre (CRUKDF 0318-FC) and Epsom Medical Research (EMR2018), with support from AstraZeneca, who were not involved in the study design, collection, analysis, interpretation of data, the writing of this article or the decision to submit it for publication.

## Conflict of Interest

The authors declare that the research was conducted in the absence of any commercial or financial relationships that could be construed as a potential conflict of interest.

## Publisher’s Note

All claims expressed in this article are solely those of the authors and do not necessarily represent those of their affiliated organizations, or those of the publisher, the editors and the reviewers. Any product that may be evaluated in this article, or claim that may be made by its manufacturer, is not guaranteed or endorsed by the publisher.
